# Burkitt’s lymphoma of the prostate presenting as acute urinary retention: a case report

**DOI:** 10.1186/s12894-020-00616-3

**Published:** 2020-05-06

**Authors:** Marcus Derigs, Anika Pehl, Jorge Riera-Knorrenschild, Rainer Hofmann, Axel Hegele

**Affiliations:** 1 Department of Urology and Pediatric Urology, University Hospital, Philipps-University Marburg, Baldingerstr. 1, 35043 Marburg, Germany; 2 Department of Pathology, University Hospital, Philipps-University Marburg, Baldingerstr 1, 35043 Marburg, Germany; 3 Department of Hematology and Oncology, University Hospital, Philipps-University Marburg, Baldingerstr. 1, 35043 Marburg, Germany

**Keywords:** Urinary retention, Burkitt’s lymphoma of the prostate, Non-Hodgkin lymphoma of the prostate

## Abstract

**Background:**

Non-Hodgkin lymphomas, which include Burkitt’s lymphoma, affect the prostate in only 0.1% of cases. They most commonly present as painless lymphadenopathy elsewhere in the body and can cause abdominal or thoracic pain and systemic symptoms such as fever, weight loss and night sweats. Here we report a rare case of sporadic Burkitt’s lymphoma of the prostate whose initial clinical presentation was acute urinary retention.

**Case presentation:**

A 28-year-old Caucasian male presented repeatedly with urinary retention. First, he was misdiagnosed with alcohol-induced urinary retention and later with benign prostatic hyperplasia. After the appearance of new symptoms, including hematuria and hydronephrosis, endoscopic and radiographic evaluation was performed. Transurethral biopsy of the prostate secured the diagnosis of Burkitt’s lymphoma. The symptoms receded under chemotherapy and complete remission of the disease was established.

**Conclusion:**

This case report brings lymphomas into focus as a differential diagnosis for urinary retention in young males. Early use of extensive diagnostic measures is advised in patients with urinary retention for uncertain reasons to make prompt diagnosis and start appropriate treatment early.

## Background

Burkitt’s lymphoma is a specific non-Hodgkin B-cell lymphoma which first was described by Denis Burkitt in 1958 [1]. It makes up less than 1% of all non-Hodgkin lymphomas but is the most common non-Hodgkin lymphoma in childhood [2].

It can be divided into three subtypes: endemic, sporadic and immunodeficiency-associated. The endemic type is associated almost always with Epstein-Barr virus (EBV) infection and occurs frequently in African children. In western countries the sporadic form is more common. The immunodeficiency-associated type is seen in patients with HIV infection [3].

Sporadic Burkitt’s lymphoma most commonly presents as abdominal lymphadenopathy with abdominal pain and other gastrointestinal symptoms [4]. It less commonly affects the head and neck region and only seldom occurs extranodally [5]. Non-Hodgkin’s lymphomas affect the prostate in only 0.1% of cases [6]. A couple of case reports exists of prostatic Burkitt’s lymphoma in childhood [7, 8]. In adults only four cases have been described [6, 9–11].

We report another case of prostatic Burkitt’s lymphoma in a young male whose initial clinical sign was urinary retention. This case report and review of the literature are indented to reintroduce Burkitt’s lymphoma and lymphomas as a whole to the list of differential diagnoses for acute urinary retention. Thereby it is supposed to sharpen diagnostic and clinical skills of urologists, oncologists and pathologists with special regard to treating young adult patients.

## Case presentation

A 28-year-old Caucasian male (80 kg, 178 cm) was admitted to the emergency room with acute urinary retention and lower abdominal pain. The patient had consumed 1 l of wine and several beers within the last few hours. Otherwise, he was in a good general condition and had not complained about any urinary symptoms beforehand. The patient did not have any pre-existing conditions and his family history was negative for genitourinary disease including renal calculi. He admitted to smoking cigarettes and drinking alcohol regularly for more than 10 years. He did not take any illicit drugs or prescription medication. He had no known allergies. Physical examination revealed a distended lower abdomen. Digital rectal examination and all other physical findings did not show any abnormalities. Neurological examination was non-contributory. Ultrasound showed normal kidneys and a full bladder without any wall irregularities. The prostate was unremarkable and its size was not documented. Hence, the first working diagnosis was alcohol-induced urinary retention. Consequently, a Foley catheter was placed without any problems. It drained 1 l of clear urine. The complaints of the patient diminished gradually. Urinalysis showed normal values. A therapy trial with the nonsteroidal anti-inflammatory drug diclofenac (75 mg, twice daily) was initiated. The patient came back 2 days later for a trial without catheter (TWOC). Hereafter, he was able to urinate sufficiently. A month later, the patient suffered acute urinary retention again, this time without being under the influence of alcohol or other drugs. Physical examination and abdominal ultrasound now both revealed an enlarged prostate measuring approximately 50 ml. Laboratory tests did not show any abnormal values, including those of prostate-specific antigen (PSA) and lactate dehydrogenase (LDH). The working diagnosis was changed to urinary retention caused by benign prostatic hyperplasia. Alpha-blocker therapy with tamsulosin (0.4 mg, once daily) was started. Three days later another TWOC failed and a new Foley catheter was inserted. During the following weeks intermittent vesical tenesmus developed. Symptomatic treatment with the muscarinic antagonist trospium chloride (15 mg, twice daily) was started. Cystoscopy revealed diffusely erythematous and villous epithelium of the prostatic urethra up to the bladder neck and large obstructive lobes. An appointment was arranged for a transurethral resection of the prostate and bladder in a month’s time. In the meantime, the patient developed persistent right-sided flank pain. Urinalysis showed leukocyturia and right-sided pyelonephritis was diagnosed. Antibiotic treatment with ciprofloxacin (500 mg, twice daily) was started by his general practitioner. Two days later the patient presented with hematuria and right-sided 2^nd^ degree hydronephrosis in the emergency room. Right-sided renal colic and hemorrhagic cystitis was suspected and the patient referred to the Department of Urology. Spasmoanalgesic therapy with metamizole (1 g, four times daily) was initiated and antibiotic treatment continued. A low-dose CT scan did not show urolithiasis but a tumor of the prostate. Cystoscopy revealed obstructive lobes of the prostate and an erythematous epithelium of the prostatic urethra and bladder neck again. The new working diagnosis was an unnoticed passing of the ureteral calculus alongside cystitis and benign prostatic hyperplasia, albeit persisting right-sided hydronephrosis. Flank pain and hematuria receded under analgesic therapy and Foley catheter placement. Transurethral biopsy of the prostate was scheduled. Until then left-sided flank pain and concomitant 2^nd^ degree hydronephrosis developed while 2^nd^ degree hydronephrosis on the right side persisted. Creatinine levels were elevated slightly and LDH levels were still within normal range. Cystoscopy now revealed a partly villous and solid tumor formation of the prostatic urethra which was infiltrating the trigone of the bladder including the ureteric orifices. Biopsies were taken endoscopically and ureteral stents were inserted bilaterally.

**Fig. 1:**
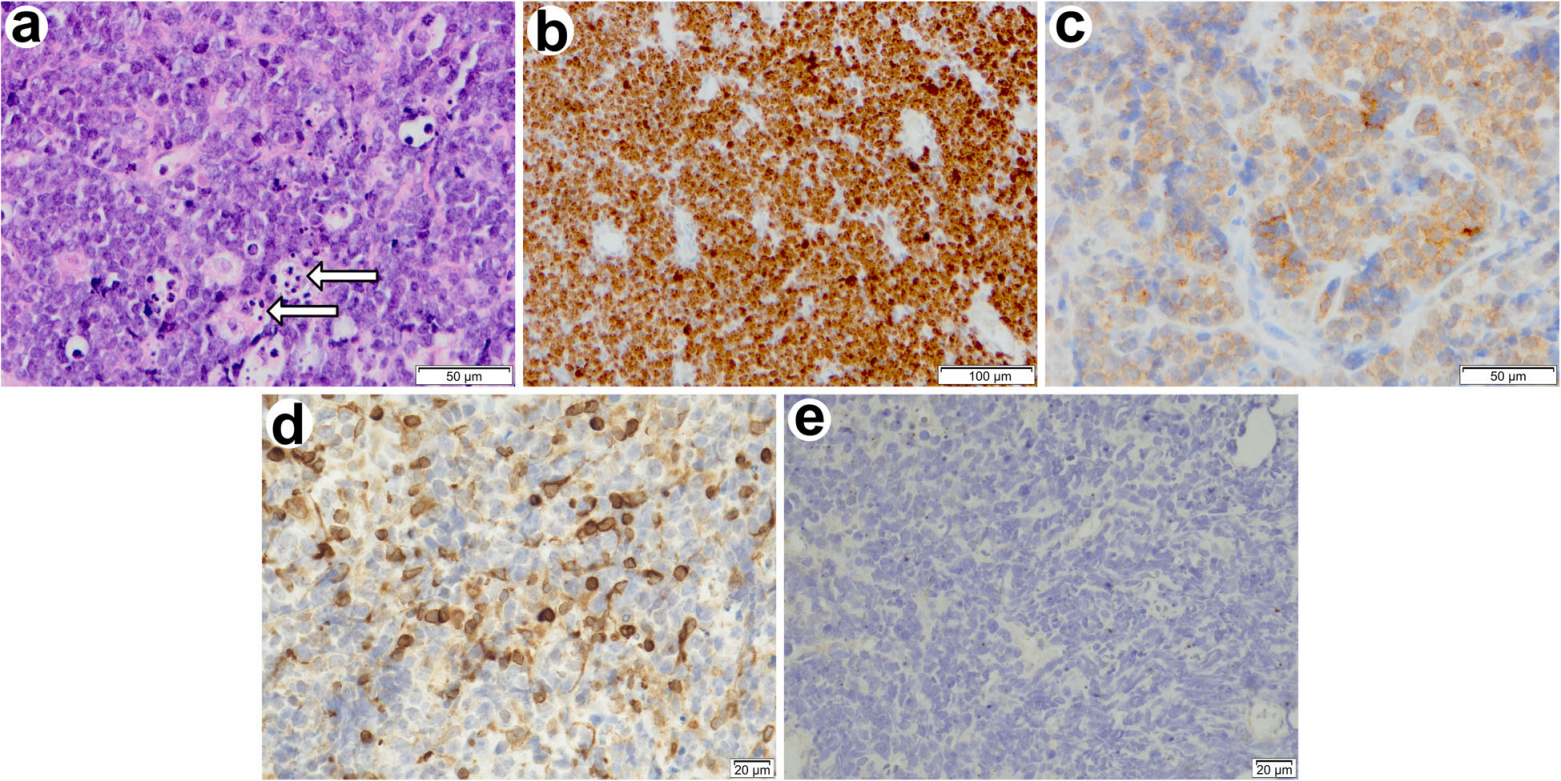
Histological and immunophenotypic features of prostatic Burkitt’s lymphoma. Microscopic observation of transurethral biopsy of the prostate and bladder neck. Homogeneously basophilic, poorly differentiated B-cells and scattered tingible body macrophages with pale cytoplasm creating the characteristic starry sky appearance **a**. Lymphoma cells are almost 100% Ki-67 positive **b**, show moderate CD10 expression **c**, atypical Bcl-2 positivity **d** and negative results for EBER **e**. **a** Haematoxylin and eosin stain (200x magnification) revealing tingible body macrophages (arrows). **b** Ki-67 reactivity (100x magnification). **c** CD10 reactivity (200x magnification). **d** Bcl-2 reactivity (400x magnification). **e** EBER in situ hybridization (400x magnification)

The histologic specimen provided evidence for a highly malignant Burkitt’s lymphoma and proved the diagnosis of benign prostatic hyperplasia wrong (Fig. 1). It showed diffuse infiltration of the prostatic parenchyma by sheets of monomorphic medium-sized lymphoid cells with basophilic nucleoli. They were highly proliferative, indicated by many mitotic figures and also had a high number of apoptotic cells. Scattered among them were numerous tingible body macrophages. Due to their pale cytoplasm with incorporated apoptotic bodies they created a so called starry sky appearance, which is characteristic for Burkitt’s lymphoma. Immunohistochemistry confirmed this diagnosis and showed the typical reactivity for CD20, a co-expression for CD10 and a strong expression for Ki-67 of nearly all tumor cells. Untypical for Burkitt’s lymphoma was the relatively strong Bcl-2 expression of B-cells, which is only expressed by about 10% of Burkitt’s lymphomas [12]. The Ebstein-Barr virus-encoded small RNAs (EBER) in situ hybridization stain was negative.

**Fig. 2:**
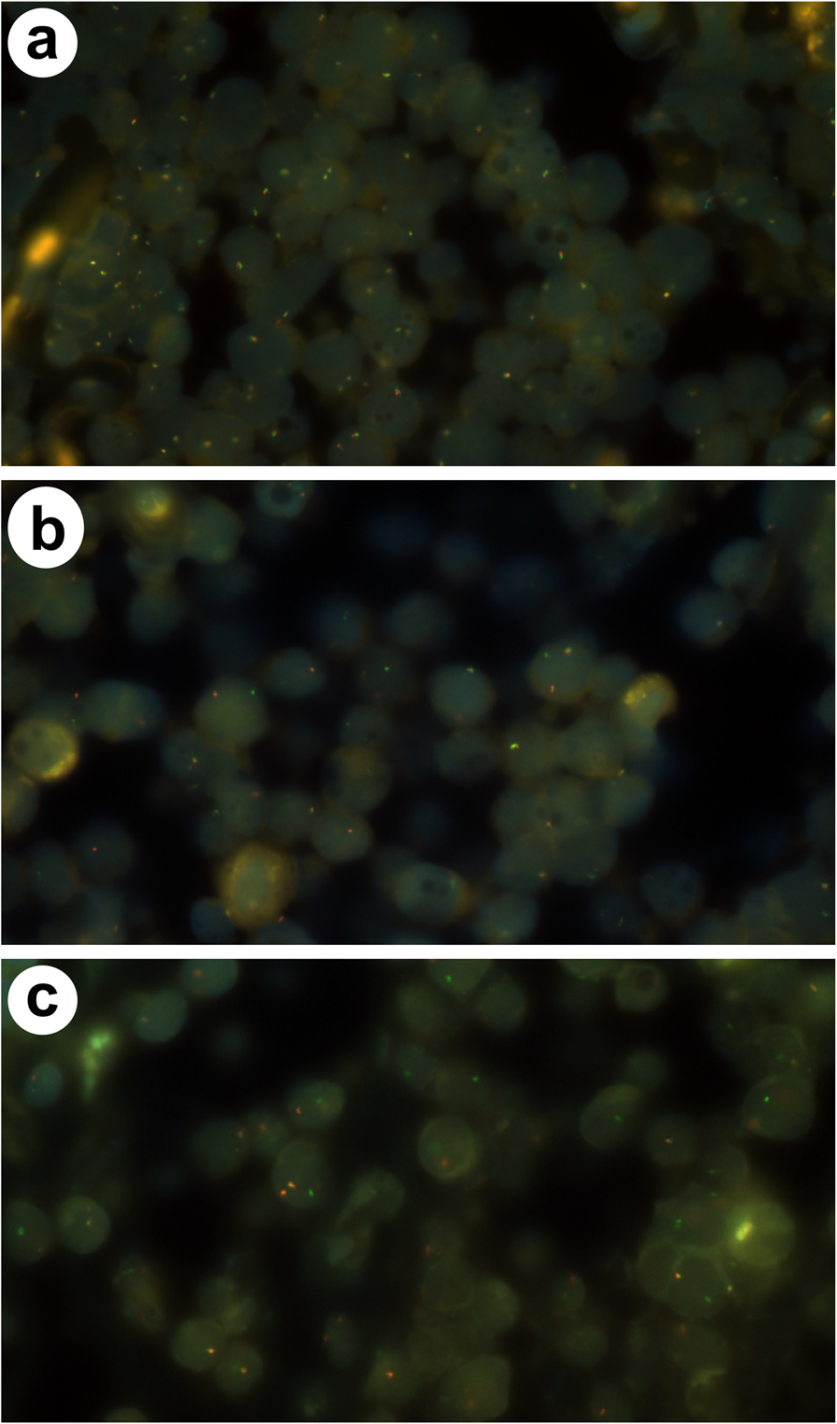
FISH analyses of prostatic Burkitt’s lymphoma. Microscopic observation of transurethral biopsy of the prostate and bladder neck. **a** FISH for *MYC*. Break apart assay to detect breakpoints in the *MYC* gene, showing separation of the probes (red and green) on one allele (1000x magnification). **b** FISH for *BCL6*. The break apart assay shows no evidence of breakpoints in the *BCL6* gene (1000x magnification). **c** FISH for t(8;14). Fusion assay confirming the typical translocation t(8;14) (1000x magnification)

Diagnosis was further confirmed by fluorescence in situ hybridization that gave normal results for the *BCL6* probe, but showed the typical translocation t(8;14) with probes for *MYC* and t (8;14) (Fig. 2). This translocation induces the fusion of *MYC* and *IGH* gene loci and thereby leads to the dysregulation of the protooncogene *MYC* [13].

**Fig. 3:**
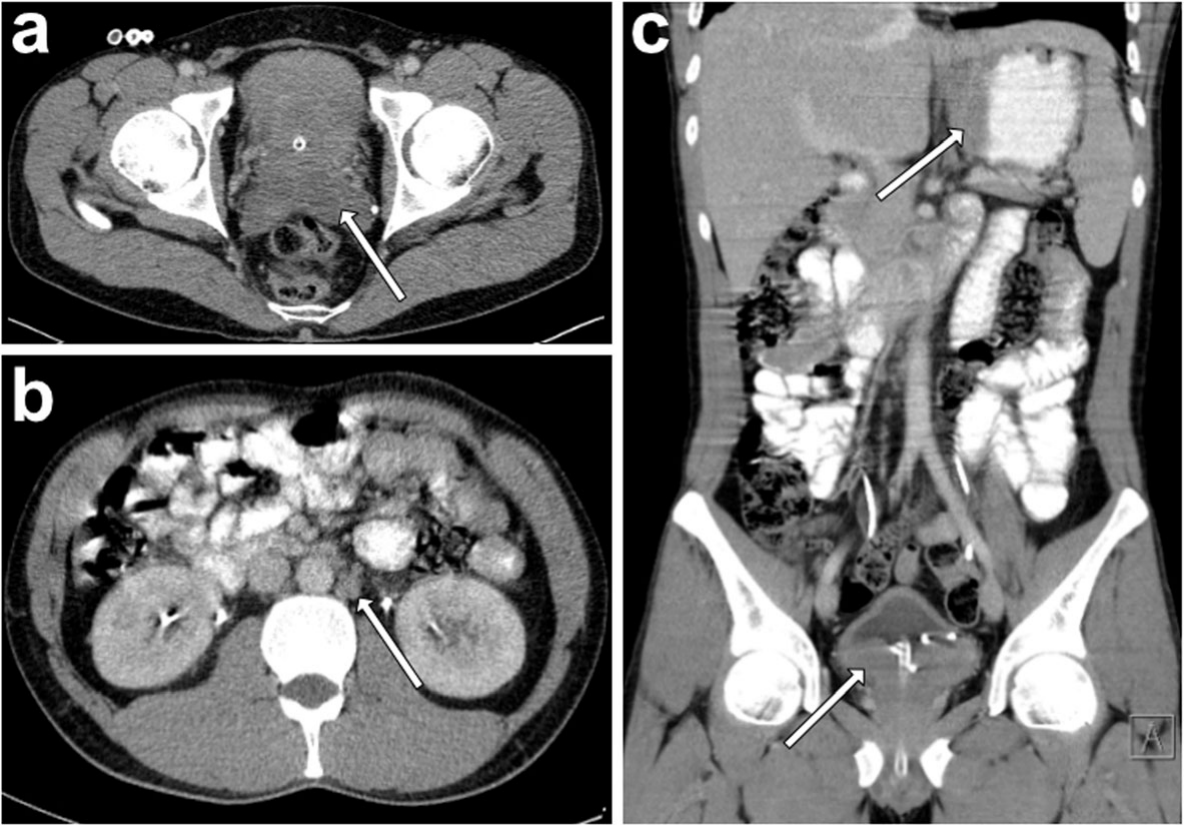
Radiographic extent of Burkitt’s lymphoma. Contrast enhanced staging CT scan after diagnosis of prostatic Burkitt’s lymphoma. **a** Axial view showing prostatic involvement and infiltration of the seminal vesicles (arrow). **b** Axial view showing involvement of paraaortic lymph nodes (arrow). **c** Coronal view showing bladder infiltration and gastric involvement (arrows). Ureteral stents and Foley catheter are visible

The patient was referred to the Department of Oncology and was scheduled for staging CT scan in preparation for chemotherapy 1 week later. His general condition was progressively declining as he experienced night sweats, melena and constipation. Laboratory results showed anemia (8.2 g/dl), increased C-reactive protein (CRP) levels and normal LDH levels. Contrast-enhanced staging CT of thorax and abdomen revealed an 85 × 65 × 44 mm tumor of the prostate with seminal vesicle and bladder invasion. Also, retroperitoneal and iliac chain lymphadenopathy (up to 17 × 13 mm in size) and gastric wall thickening were present (Fig. 3).

Gastroscopy ruled out upper gastrointestinal bleeding and confirmed diagnosis of gastric involvement of Burkitt’s lymphoma via biopsy. Bone marrow biopsy and lumbar puncture excluded involvement of bone marrow and liquor concluding a stage IV Burkitt’s lymphoma according to the Lugano staging system [14]. Before chemotherapy was initiated, the patient performed sperm cryopreservation.

**Fig. 4:**
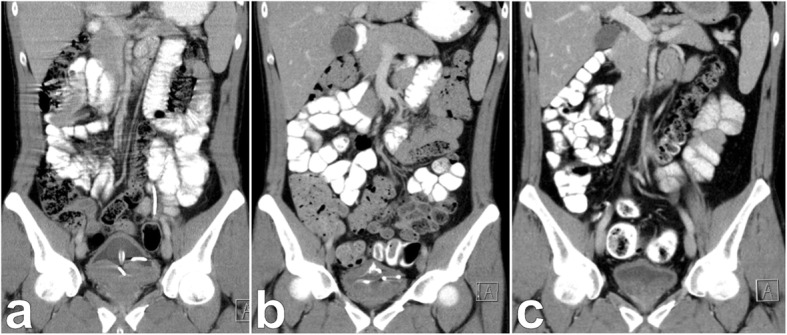
Regression of prostatic Burkitt’s lymphoma during chemotherapy. Coronal view of contrast enhanced CT scan showing prostatic Burkitt’s lymphoma before (**a**), during (**b**) and after (**c**) chemotherapy. Burkitt’s lymphoma caused urinary retention and bilateral hydronephrosis. **a** Foley catheter and ureteral stents were inserted and chemotherapy started. **b** After one of 6 cycles significant downsizing of the lymphoma was seen. **c** Foley catheter and ureteral stents could be removed after 6 cycles of chemotherapy

Ultimately, the diagnosis of Burkitt’s lymphoma was made 4 months after initial presentation and only 6 days later systemic chemotherapy according to the GMALL (German Multicenter Study Group for Adult Acute Lymphoblastic Leukemia) B-ALL/NHL 2002 protocol was started. It consisted of a total of 6 cycles of 3 different drug regimen including dexamethasone, cyclophosphamide, rituximab, dexamethasone, vincristine, ifosfamide, etoposide, cytarabine and high-dose methotrexate. Concurrent medication was composed of acyclovir, trimethoprim/sulfamethoxazole, ciprofloxacin, pantoprazole, G-CSF, amphotericin B mouth rinse and Glandomed® mouthrinse. The first cycle was given as a milder regimen with dexamethasone and cyclophosphamide together with hydration, allopurinol and urinary alkalization in order to prevent tumor lysis syndrome. Nonetheless, the patient developed neutropenic fever and grade 4 mucositis and required extensive analgesic and antibiotic treatment. Due to increasing abdominal pain a CT scan was done which excluded a tumor lysis with gastric perforation. It rather showed a response to treatment according to Lugano treatment response criteria (Fig. 4) [14].

During the following cycles there was one more episode of neutropenic fever. Mucositis persisted only on a lower level. The Foley catheter was removed 2 months after initiation of chemotherapy. Hereafter satisfactory voiding with insignificant post-void residual volume was established. After finishing chemotherapy, a CT scan showed complete response. Following this, both ureteral stents were removed. Subsequent sonography of the kidneys could exclude persisting hydronephrosis. Another CT scan 3 months later confirmed complete remission of Burkitt’s lymphoma (Fig. 4c). The patient recovered completely and is now participating in follow-up care.

## Discussion and conclusion

Acute urinary retention (AUR) is one of the most common urologic emergencies and will be encountered by physicians of every specialty. It is described as the sudden inability to urinate and is usually managed by transurethral catheterization [15]. The incidence of AUR is reported in 2.2 events per 1000 man-years in men over 45 years old [16]. Almost all patients with AUR are men at the age of over 60 with an identifiable predisposing risk factor. In general, there are three reasons for AUR. Firstly, any event that increases the resistance to subvesical urinary flow. This is mainly achieved by mechanical obstruction by a variety of reasons (Table 1). The most common cause is forward displacement of the internal urethral orifice by benign prostatic hyperplasia (BPH) in men above the age of 50.

**Table 1:** Reasons for urinary retention. Modified after [18, 25]

**Reasons for mechanical obstruction**	
Adenocarcinoma or sarcomatoid carcinoma of the prostate	
Benign prostatic hyperplasia	
Prostatic, appendiceal abscess	
Prostatitis, cystitis, urethritis	
Chronic lymphocytic leukemia	
Sarcoma, lymphoma of the prostate	
Urolithiasis	
Ectopic Ureterocele	
Constipation	
Urethral valves, strictures	
Uterine fibroids or other tumors	
Impacted retroverted uterus	
Gastrointestinal tumors	
Haematocolpos	
Cystocele or rectocele	
Foreign bodies (external or internal)	
Trauma	
Phimosis, paraphimosis	
Blood clots from bleeding in the bladder or upper urinary tract	
**Reasons for increased sphincter tone**	
Neurofibromatosis in the prostate	
Anorectal surgery	
Fowler’s syndrome	
**Reasons for interference with sensory or motor innervation to the bladder**	
Diabetic cystopathy	
Herpes zoster in the sacral dermatomes (S2-S4)	
Drug-induced	
Cauda equina syndrome/Elsberg syndrome/Guillan-Barré syndrome	
Psychogenic	
Transverse myelitis in Lyme disease	

However, AUR is a rare event in young adults. In this age group the most common cause for AUR are urethral calculi, urethral strictures or urinary tract infections such as cystitis, urethritis or prostatitis [17, 18]. Hence, younger patients have to be carefully evaluated. Only very rarely BPH leads to acute urinary retention in young adult males below the age of 40. A few case reports exist of such patients as well as for children [19–21]. Other reasons causing obstruction are pelvic and prostatic tumors of any kind. Also, a dynamic obstruction due to an increase in the smooth and/or striated muscle tone has to be considered. Secondly, interrupted sensory innervation of the bladder wall or motor supply of the detrusor muscle is a reason for AUR. This might be caused by demyelination or spinal cord compression [22]. The depression of the cortical areas for voluntary micturition or of the detrusor nuclei in the brainstem and interference of neurotransmission at the bladder or urethra by a number of drugs can also cause AUR [23]. Frequent triggers are central nervous depressants such as alcohol and medication like anticholinergics, sympathomimetics and antineoplastic agents [24].

Thirdly, AUR is precipitated by any situation that leads to an overdistension of the bladder (post-surgery, drugs) [25].

The reason for obstructive urinary retention in the presented case turned out to be prostatic Burkitt’s Lymphoma, a subtype of non-Hodgkin lymphoma. Less than 0.1% of all non-Hodgkin lymphomas affect the prostate of which diffuse large B-cell lymphoma is the most common entity [6]. In younger aged patients malignant rhabdomyosarcomas are the most common prostatic malignancy [26]. Only a couple of case reports exists of prostatic Burkitt’s lymphoma in childhood [7, 8]. To date in adults only four cases have been described [6, 9–11]. Lymphoma of the prostate is very rare and is usually not considered in patients with urinary retention. On the other hand, adenocarcinoma of the prostate or BPH are equally rare entities in young males and should not be assumed without further investigation either. In this case a young male was treated for urinary retention and was misdiagnosed twice. Initially, the consumption of alcohol by the patient and a lack of other findings led to the working-diagnosis of alcohol-induced urinary retention. Even though the pathophysiologic reasoning for alcohol-induced urinary retention are CNS-depression and bladder overdistention, an anti-inflammatory therapy with diclofenac was started. In the beginning, the diagnosis was affirmed by a successful TWOC, so no other diagnostic tests were performed. More common causes for AUR in this age group were not suspected due to absent flank pain, normal urinalysis and unremarkable micturition in the past. After urinary retention recurred, clinical and sonographic examination revealed an enlarged prostate. Notwithstanding the rarity in this age group, BPH was diagnosed without further workup. Prompt performance of cystoscopy or other imaging techniques would have led to the correct diagnosis earlier. Instead, progression of the lymphoma caused both-sided hydronephrosis, hematuria and systemic symptoms.

In conclusion, this case shows that the unlikely event of urinary retention in a young adult male has to be of great concern. After all, it can be the first manifestation of a serious prostatic malignancy. Thorough interviewing of previous and current symptoms and physical examination have to be performed to narrow down differential diagnoses. If benign reasons are ruled out or if initial treatment fails, more extensive diagnostic tests including cystoscopy and imaging techniques like CT or MRI scan are mandatory. Subsequent biopsy of the prostate should be performed urgently to secure the diagnosis. In our case systemic chemotherapy led to complete remission and possibly cure of the Burkitt’s lymphoma.

## Data Availability

The datasets used and/or analyzed during the current study are available from the corresponding author on reasonable request. All data generated or analyzed during this study are included in this published article.

## Abbreviations

AUR: Acute urinary retention
Bcl-2: B-cell Lymphoma 2
BPH: Benign prostatic hyperplasia
CRP: C-reactive protein
CT: Computed tomography
G-CSF: Granulocyte-colony stimulating factor
GMALL: German Multicenter Study Group for Adult Acute Lymphoblastic Leukemia
LDH: Lactate dehydrogenase
HIV: Human immunodeficiency virus
FISH: Fluorescence in situ hybridization
MRI: Magnetic resonance imaging
PSA: Prostate-specific antigen
TWOC: Trial without catheter
